# Mid-Term Exposure to Air Pollution and Acute Kidney Injury Incidence: A 10-Year Study in Eastern Poland

**DOI:** 10.3390/jcm15134929

**Published:** 2026-06-25

**Authors:** Adam Gryko, Anna Kurasz, Jolanta Małyszko, Sławomir Dobrzycki, Łukasz Kuźma

**Affiliations:** 1 Department of Invasive Cardiology, Medical University of Bialystok, M. Sklodowskiej-Curie 24a, 15-276 Bialystok, Poland; 2 Department of Nephrology, Dialysis and Internal Medicine, Medical University of Warsaw, 02-091 Warsaw, Poland; 3 Liverpool Centre for Cardiovascular Science, University of Liverpool, Liverpool John Moores University and Liverpool Heart & Chest Hospital, Liverpool L3 5AH, UK; 4 Department of Cardiac Surgery and Transplantology, National Medical Institute of the Ministry of the Interior and Administration, 02-507 Warsaw, Poland

**Keywords:** air pollution, particulate matter, acute kidney injury, renal function, exposome

## Abstract

**Background/Objectives:** Air pollution is associated with many adverse health consequences, including deteriorated kidney function. The aim of the research was to determine the association of medium-term exposure to air pollutants and hospitalizations due to acute kidney injury (AKI). **Methods**: The retrospective population-based study was conducted on the EP-PARTICLES cohort between 2011 and 2020 (80,000,000 person-years). We estimated municipality-specific associations between air pollution and AKI admissions using generalized additive models with Poisson regression. Results are reported as risk ratio in AKI admissions (RR) with corresponding 95% confidence intervals (95% CI). **Results**: During the 10-year study period, 47,467 AKI cases were reported (median age 77 years, IQR 68–84; 51.2% women). Mean concentrations of pollutants were 21.4 µg/m^3^ (SD 5.2) for particulate matter with a diameter of 2.5 μm or less (PM_2.5_), 7.5 µg/m^3^ (1.8) for nitrogen dioxide (NO_2_), and 1.8 ng/m^3^ (0.8) for benzo[a]pyrene (BaP). In mid-term exposure analyses (lag 0–30), each 10 µg/m^3^ increase in PM_2.5_, PM_10_, NO_2_ and CO, and each 1 µg/m^3^ increase in BaP, was associated with higher AKI risk, with the strongest effect observed for NO_2_ (RR 1.066, 95% CI 1.033–1.099). No association was found for SO_2_. Subgroup analyses showed consistent directions of association across sex and age groups, with NO_2_ remaining the most detrimental pollutant. Although statistical significance varied between pollutants, no significant effect modification by sex or age was observed (*p* > 0.05). **Conclusions**: Mid-term exposure to ambient air pollution is associated with an increased risk of AKI-related hospitalizations, with NO_2_ showing the strongest effects. These findings identify mid-term exposure as a relevant temporal window and support the role of air pollution as a modifiable risk factor for AKI.

## 1. Introduction

Over the past decades, air pollution has been consistently associated with a wide range of adverse health outcomes, being the most significant environmental threat to global public health. As indicated in the Global Burden of Disease Study 2023, air pollution was the second most important risk factor responsible for Disability Adjusted Life Years (DALYs), after hypertension, overtaking smoking, high body-mass index or impaired fasting glucose [1]. According to the European Environment Agency, in 2022, polluted air was responsible for 239,000 deaths in Europe [2]. It is estimated that over 90% of the global population lives in areas where air pollution levels exceed recommended limits [3].

Air pollutants affect multiple organs through a variety of mechanisms. Inhaled particulate matter induces the release of inflammatory mediators, leading to both pulmonary and systemic inflammation [4]. Moreover, exposure to ambient air pollutants has been shown to disrupt autonomic nervous system function and to promote oxidative stress [5,6,7]. In addition, airborne particulates may translocate across the pulmonary epithelium into the bloodstream, thereby exerting direct effects on distant tissues [8]. These processes contribute to endothelial dysfunction, vascular injury, and dysregulation of microcirculatory perfusion, which may be particularly relevant for highly vascularized organs such as the kidneys.

For years, research has predominantly focused on the association between air pollution and pulmonary as well as cardiovascular diseases [9,10,11,12]. However, an increasing body of evidence has recently emerged regarding the impact of air pollution on renal function. Kidney diseases, particularly chronic kidney disease (CKD), represent a major global public health challenge. In 2017, CKD affected approximately 9.1% of the global population [13], while in the United States, the prevalence among adults is estimated at around 14% [14]. Beyond CKD, acute kidney injury (AKI) constitutes a clinically significant condition associated with increased morbidity, mortality, and healthcare burden, yet its environmental determinants remain insufficiently explored [15].

The majority of existing studies examining the relationship between air pollution and kidney disease have focused on long-term exposure [16,17,18,19], whereas only a limited number have addressed the effects of short-term impact on renal function [20,21]. However, short-term exposure windows may not adequately reflect the cumulative biological burden of air pollution, particularly in the context of conditions such as AKI, which likely develop over a longer exposure period rather than as a consequence of abrupt day-to-day changes. Furthermore, most of this research has been conducted in large urban centers and highly industrialized regions, with a substantial proportion originating from Asia and the United States [22]. In contrast, relatively few studies have been performed in Europe, and only one has been conducted in Central and Eastern Europe [23]. This geographic imbalance limits the generalizability of existing findings, particularly to regions with different environmental profiles and healthcare systems.

Therefore, the aim of our study was to investigate the association between mid-term exposure to air pollution and hospital admissions due to AKI in eastern Poland over the years 2011–2020.

## 2. Methods

### 2.1. Study Design and Data Source

The research is part of the EP-PARTICLES project, details of which have been presented in earlier publications [24,25,26,27,28]. The study was conducted in a region of eastern Poland that is characterized by specific socioeconomic and demographic features, including relatively low industrial activity, limited urbanization, and lower gross domestic product (GDP) per capita compared with Western European regions. The area is predominantly rural, with low population density. Geographically, the analyzed region is situated in the eastern part of Poland and forms part of the European Union’s external border, adjoining Belarus, Ukraine, Lithuania, and the Russian Federation. The analysis covered five administrative regions (voivodeships): Podlaskie, Warmińsko-Mazurskie, Lubelskie, Podkarpackie, and Świętokrzyskie. These regions together include 101 counties and 709 municipalities, with a total population of approximately 8 million residents. The study period spanned from 2011 to 2020, corresponding to nearly 80 million person-years of observation.

Information on hospital admissions due to AKI was retrieved from the database of the National Health Fund and identified using the ICD-10 classification (code N17). All eligible hospitalizations recorded within the study area and timeframe were included in the analysis.

Environmental exposure data were obtained for key air pollutants, including PM_2.5_, PM_10_, nitrogen dioxide (NO_2_), sulfur dioxide (SO_2_), carbon monoxide (CO), and benzo[a]pyrene (BaP). Data were sourced from the Chief Inspectorate for Environmental Protection and subsequently processed to estimate exposure levels during the 30-day period preceding each hospitalization event. To enhance the spatial accuracy of exposure assessment, air pollution data were modeled at high resolution using the GEM-AQ (Global Environmental Multiscale Air Quality) model [29,30,31], in collaboration with a National Research Institute. The data on weather conditions were obtained from the Institute of Meteorology and Water Management. We used the daily mean of temperature, humidity, and atmospheric pressure obtained on local administrative units (LAU-2) level.

A 30-day exposure window was selected to capture cumulative subacute effects of air pollution while maintaining temporal proximity to the outcome and ensuring consistency with previous EP-PARTICLES analyses.

Individual-level linkage between environmental exposure and hospitalization records was achieved through spatial assignment based on postal code information, allowing integration with both air quality and meteorological datasets. This methodological approach has been widely applied and validated in prior epidemiological studies using real-world data [32,33].

### 2.2. Statistical Analysis

In the first stage, we estimated municipality-specific associations between air pollutant concentrations and AKI incidence at the LAU-2 level. Daily counts of AKI admissions were analyzed using count regression methods.

Model selection was guided by standard diagnostic procedures. Overdispersion was assessed by comparing the Pearson χ^2^ statistic to its degrees of freedom and by examining the ratio of the residual deviance to the residual degrees of freedom. Serial correlation in model residuals was evaluated using autocorrelation function (ACF) plots and the Durbin–Watson test. As evidence of overdispersion and autocorrelation was minimal, Poisson regression estimated by quasi-maximum likelihood with robust (sandwich) variance estimation was selected as the primary modeling approach.

All models were estimated at the municipality level, linking exposure and outcome data via postal codes. Short-term exposure was modeled using moving averages ranging from 0 to 30 days, defined as the mean concentration on the index day and the preceding 29 days.

The main models adjusted for ambient temperature, relative humidity, and atmospheric pressure, as well as day of the week, public (bank) holidays, influenza seasons, and the SARS-CoV-2 pandemic period. Seasonality and long-term temporal trends were controlled for using cubic spline functions of calendar time.

In the second stage, municipality-specific estimates for air pollution were pooled to obtain national-level effects using random-effects meta-regression. Between-municipality variance was estimated using the restricted maximum likelihood (REML) method, and statistical heterogeneity was quantified using the I^2^ statistic.

Effect modification was examined through stratified analyses by sex and age group. To formally test differences between subgroup-specific effect estimates (e.g., males vs. females, older vs. younger populations), *p*-values for differences in risk ratios were calculated using the Altman and Bland method. Adjustment for multiple comparisons was performed using the Bonferroni correction, and corrected *p*-values were used to determine statistical significance where applicable.

Results are reported as risk ratio in AKI admissions (RR) with corresponding 95% confidence intervals (95% CI). All statistical analyses were conducted using Microsoft Excel (version 16.78.3; Microsoft, Redmond, WA, USA) and Stata Statistical Software (version 18; StataCorp, College Station, TX, USA).

## 3. Results

During the 10-year period in the analyzed area, AKI was reported in 47,467 cases. The median age of the population was 77 years (IQR 68–84), with the majority being women (51.2%). The study region covers approximately 99,050 km^2^ and is inhabited by more than 8 million people. An analysis of air quality data at the county level showed that the daily guideline values recommended by the World Health Organization were exceeded on 58% of days for PM_2.5_ and on 4.2% of days for NO_2_. Throughout the entire ten-year study period, the annual mean concentrations of PM_2.5_ and BaP remained consistently above the limits established by the World Health Organization and the European Environment Agency. The average concentration over this period was 21.4 µg/m^3^ (SD = 5.2) for PM_2.5_, 28.46 (SD = 5.7) for PM_10_, 7.5 µg/m^3^ (SD = 1.8) for NO_2_, 273.6 µg/m^3^ (SD = 15.4) for CO, 3.6 µg/m^3^ (SD = 1) for SO_2_ and 1.8 ng/m^3^ (SD = 0.8) for BaP. Over the study period, annual mean concentrations of PM_2.5_, NO_2_, SO_2_, and BaP showed a general downward trend, whereas PM_10_ concentrations increased. The largest relative decreases were observed for BaP (7.1 to 1.2 ng/m^3^) and SO_2_ (5.8 to 2.1 μg/m^3^), while PM_2.5_ declined by approximately 44%. In contrast, PM_10_ increased by approximately 78% between 2011 and 2020. CO concentrations remained relatively stable, with no clear temporal trend (Table 1 and Table 2).

**Figure 1:**
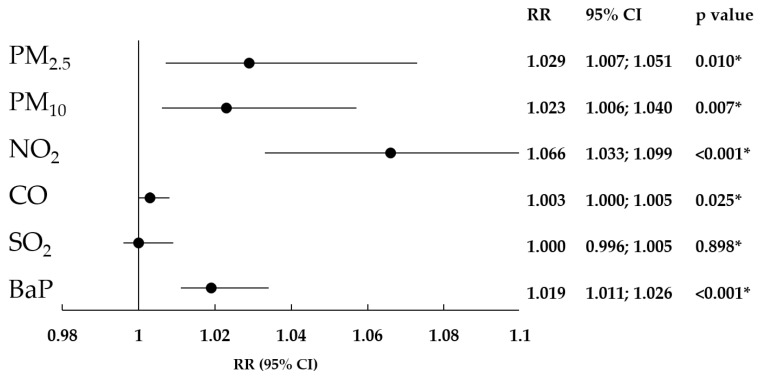
Association between air pollutants and acute kidney injury. Statistically significant results are marked with *. Abbreviations: BaP, Benzo(a)pyrene; NO_2_, nitrogen dioxide; PM_2.5_, particulate matter with a diameter of 2.5 μm or less; PM_10_, particulate matter with a diameter of 10 μm or less; SO_2_, sulfur dioxide; RR, risk ratio; CI, confidence interval.

Each 10 µg/m^3^ increment in PM_2.5_ (RR 1.029, 95% CI 1.007–1.051), PM_10_ (RR 1.023, 95% CI 1.006–1.04), NO_2_ (RR 1.066, 95% CI 1.033–1.099), CO (RR 1.003, 95% CI 1.0–1.005) concentration and 1 µg/m3 increase in BaP (RR 1.019, 95% CI 1.011–1.026) concentration was associated with an increase in AKI incidence in the mid-term exposure (lag 0–30), with the strongest effects observed for NO_2_ (RR 1.066, 95% CI 1.033–1.099). There was no association between exposure to SO_2_ and the risk of AKI (Figure 1). 

### Age and Sex Differences

**Figure 2:**
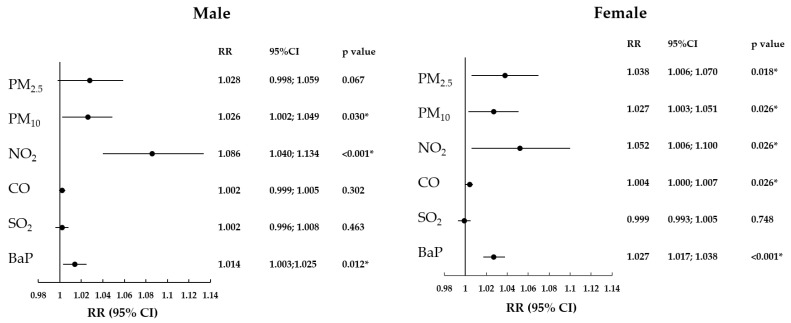
Sex-stratified associations between air pollutants and acute kidney injury over the 0–30-day lag period. Statistically significant results are marked with *. Abbreviations: BaP, Benzo(a)pyrene; NO_2_, nitrogen dioxide; PM_2.5_, particulate matter with a diameter of 2.5 μm or less; PM_10_, particulate matter with a diameter of 10 μm or less; SO_2_, sulfur dioxide; RR, risk ratio; CI, confidence interval.

**Figure 3:**
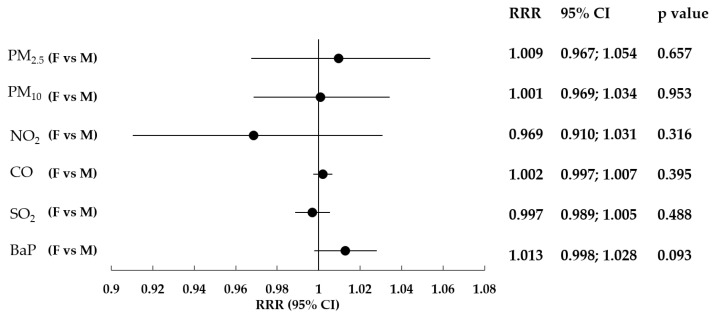
Sex differences in the association between air pollutants and acute kidney injury over the 0–30-day lag period. Abbreviations: BaP, Benzo(a)pyrene; NO_2_, nitrogen dioxide; PM_2.5_, particulate matter with a diameter of 2.5 μm or less; PM_10_, particulate matter with a diameter of 10 μm or less; SO_2_, sulfur dioxide; RR, risk ratio; CI, confidence interval; F, female; M, male.

In sex-stratified analyses, NO_2_ was significantly associated with AKI in both men (RR 1.086, 95% CI 1.040–1.134) and women (RR 1.052, 95% CI 1.006–1.100). Among men, significant associations were also observed for PM_10_ (RR 1.026, 95% CI 1.002–1.049) and BaP (RR 1.014, 95% CI 1.003–1.025), whereas PM_2.5_, CO and SO_2_ were not significantly associated with AKI. In women, the impact of PM_2.5_ (RR 1.038, 95% CI 1.006–1.070), PM_10_ (RR 1.027, 95% CI 1.003–1.051), CO (RR 1.004, 95% CI 1.000–1.007), and BaP (RR 1.027, 95% CI 1.017–1.038) on AKI incidence was observed, while no effect of SO_2_ was noted. However, in direct comparisons, no significant differences between sexes were observed (Figure 2 and Figure 3).

**Figure 4:**
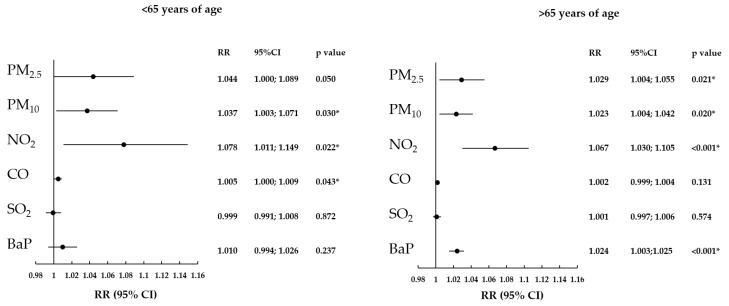
Age-stratified associations between air pollutants and acute kidney injury over the 0–30-day lag period. Statistically significant results are marked with *. Abbreviations: BaP, Benzo(a)pyrene; NO_2_, nitrogen dioxide; PM_2.5_, particulate matter with a diameter of 2.5 μm or less; PM_10_, particulate matter with a diameter of 10 μm or less; SO_2_, sulfur dioxide; RR, risk ratio; CI, confidence interval.

**Figure 5:**
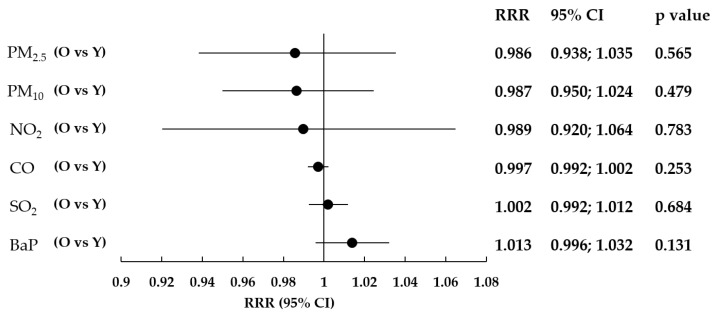
Age differences in the association between air pollutants and acute kidney injury over the 0–30-day lag period (≥65 y.o. vs. <65 y.o.). Abbreviations: BaP, Benzo(a)pyrene; NO_2_, nitrogen dioxide; PM_2.5_, particulate matter with a diameter of 2.5 μm or less; PM_10_, particulate matter with a diameter of 10 μm or less; SO_2_, sulfur dioxide; RR, risk ratio; CI, confidence interval; O, ≥65 years old; Y, <65 years old.

Across both age groups (over and under 65 years old), NO_2_ emerged as the most detrimental pollutant linked to AKI incidence, with comparable effect estimates in individuals <65 years (RR 1.078, 95% CI 1.011–1.149) and ≥65 years (RR 1.067, 95% CI 1.030–1.105) In people <65 years old, increased AKI incidence was observed in relation to PM_2.5_ (RR 1.044, 95% CI 1.000–1.089), PM_10_ (RR 1.037, 95% CI 1.003–1.071), NO_2_ and CO (RR 1.005, 95% CI 1.000–1.009), whereas no association was found for BaP or SO_2_ exposure. Among older individuals, PM_2.5_ (RR 1.029, 95% CI 1.004–1.055), PM_10_ (RR 1.023, 95% CI 1.004–1.042), NO_2_ and BaP (RR 1.024, 95% CI 1.003–1.025) showed significant relationships with AKI, while CO and SO_2_ remained non-significant. Despite these patterns, direct comparison between age groups did not reveal significant differences (Figure 4 and Figure 5).

## 4. Discussion

To our knowledge, this is the first study providing a comprehensive assessment of mid-term exposure to six major air pollutants, including BaP, in relation to AKI hospitalisations in a large-scale European population. We demonstrate that exposure within a 30-day window is associated with a significantly increased risk of AKI, with NO_2_ showing the strongest and most consistent effect across all analyses.

Our findings should be interpreted within the broader framework of the exposome, originally proposed by Dr. Christopher Wild as a comprehensive representation of all environmental exposures across the life course, encompassing external exposures (e.g., air pollution, chemicals, climate), internal biological responses, and their dynamic interactions [34]. This paradigm shifts the focus from isolated risk factors toward the cumulative and synergistic effects of environmental influences on disease development [35]. An increasing body of evidence indicates that the exposome is highly relevant to kidney health. Environmental exposures such as heavy metals, including cadmium and lead, have been consistently associated with tubular injury and accelerated decline in kidney function [36]. In parallel, climate-related factors—particularly heat stress [37] and recurrent dehydration—have emerged as important contributors to kidney injury through mechanisms involving hypoperfusion and inflammation.

Within this framework, air pollution represents a major component of the external exposome. A growing body of epidemiological evidence supports its association with AKI across different exposure windows. Large-scale cohort studies have demonstrated that long-term exposure to air pollution increases the risk of incident AKI. In a nationwide study of over 61 million Medicare beneficiaries, chronic exposure to PM_2.5_ and NO_2_ was associated with a higher risk of first AKI hospitalization, with effects persisting even below current regulatory thresholds [38]. Complementary evidence from short-term exposure studies indicates that acute increases in pollutant concentrations may act as immediate triggers of kidney injury. Case-crossover analyses from New York State [21] and South Korea [39] have shown that transient elevations in PM_2.5_, NO_2_, and O_3_ are associated with increased risk of AKI events and emergency department visits, particularly among individuals with pre-existing comorbidities. Moreover, Lopez et al. observed that heat was the environmental factor with the greatest short-term impact on hospitalizations due to AKI, while the most harmful pollutant was NO_2_, which is in line with our results [40]. To date, only one study from Central and Eastern Europe, by Kuźma et al., has examined this relationship, reporting no association between short-term (weekly) NO_2_ exposure and eGFR despite observing adverse effects of PM_2.5_ [23].

Against this background, our findings provide important evidence for the role of mid-term exposure—a time window that may bridge the gap between acute triggers and chronic susceptibility. To our knowledge, only one study has assessed the effect of medium-term exposure (last 28 days) to air pollution on kidney function so far. This was a US study conducted between 1998 and 2016 [41]. In this study, an IQR increase in PM_2.5_ was associated with higher uric acid concentration (β = 0.214 mg/dL, *p* = 0.002), an IQR increase in sulfur concentration (PM_2.5_ component) was associated with a lower eGFR (−1.281 mL/min/1.73 m^2^, *p* = 0.032) and a higher risk of CKD (OR = 1.39, *p* = 0.0008), and an IQR increase in lead concentration was associated with a lower eGFR (−1.008 mL/min/1.73 m^2^, *p* = 0.049). Our study is therefore the first to assess the mid-term exposure on AKI incidence.

We demonstrate that pollutant exposure over a 30-day period is associated with increased AKI risk. Notably, NO_2_ showed the strongest association, aligning with previous studies identifying traffic-related pollution as a key determinant of kidney outcomes [40,42,43]. Exposure to NO_2_ increases oxidative stress, leads to the activation of proinflammatory cytokines, causes endothelial dysfunction and reduced NO availability, which leads to impaired renal microcirculation and tubular hypoxia, and also activates the RAA system [44,45]. The magnitude of the observed associations is comparable to those reported in short-term studies, suggesting that cumulative exposure over several weeks may have clinically meaningful effects.

The observed associations with particulate matter (PM_2.5_ and PM_10_) are consistent with prior literature, although effect sizes were smaller than for NO_2_. This may reflect differences in pollutant composition, biological activity, or exposure misclassification. Of particular interest is the association observed for BaP in this study, a polycyclic aromatic hydrocarbon and marker of combustion-related pollution—a key pollutant associated with Polish Smog [46,47]. While BaP has been relatively understudied in the context of kidney outcomes, its known genotoxic and pro-oxidative properties suggest a plausible role in renal injury, potentially through DNA damage, epigenetic alterations, and disruption of cellular homeostasis [48]. In a mouse model, short-term BaP exposure induced a rapid increase in serum creatinine within 3 days, accompanied by oxidative stress and subsequent inflammatory and apoptotic injury [48]. These findings highlight the importance of considering a broader spectrum of pollutants beyond routinely studied components.

The biological plausibility of our results is supported by multiple mechanistic pathways. Air pollution exposure induces oxidative stress, systemic inflammation, endothelial dysfunction, and alterations in vascular tone, all of which may impair renal microcirculation and promote tubular injury [4,49,50]. Recent multi-omics analyses further suggest that pollutant exposure may influence key molecular regulators, including IL18, KLF2 (Kruppel-like Factor 2), NPPA (Natriuretic Peptide Precursor A), and TGIF1 (Transforming growth factor β-induced factor homeobox 1), affecting immune responses, endothelial function, and cellular metabolism [51]. These processes appear to predominantly affect proximal tubular cells, endothelial cells, and immune pathways, providing mechanistic support for the observed epidemiological associations. Importantly, air pollution may not only act as an independent risk factor but also amplify the effects of established AKI determinants, including hypertension and diabetes, thereby further increasing susceptibility to AKI [49].

Subgroup analyses demonstrated a consistent direction of associations across sex and age categories, with NO_2_ remaining the most detrimental pollutant. Although statistical significance varied between pollutants within subgroups, formal tests for interaction did not indicate significant effect modification. These findings suggest that the impact of air pollution on AKI risk is not limited to specific demographic groups but rather reflects a broadly shared susceptibility at the population level.

Previous studies have typically focused on large urban centers and highly industrialized regions, with most of them coming from Asia and the United States [38,42,52,53,54]. A notable aspect of our study is its focus on a region of Eastern Europe generally considered to have relatively lower levels of air pollution compared to highly urbanized regions. Despite this, we observed consistent associations between pollutant exposure and AKI risk. This finding aligns with emerging evidence indicating that adverse health effects occur even at concentrations below current air quality standards [24,25], raising concerns about the adequacy of existing regulatory thresholds.

This study has several limitations. First, due to its retrospective and observational design, causal inference cannot be established, and the observed associations should be interpreted with caution. However, the large-scale, population-based nature of the cohort, covering over 80 million person-years, strengthens the robustness and generalizability of the findings. Second, despite adjustment for multiple factors, residual confounding cannot be excluded. Individual-level data on important variables such as smoking status, diet, occupational exposures, time-activity patterns (e.g., time spent indoors or commuting), medication use (e.g., nephrotoxic drugs), and comorbidities were not available and may have influenced both exposure and outcome. Third, exposure assessment was based on modeled ambient air pollution concentrations rather than individual-level measurements, and AKI cases were identified using administrative data (ICD-10 codes). Both outcome ascertainment based on ICD-10 coding and exposure assignment using municipality-level residential centroids may have introduced non-differential misclassification, which would be expected to bias the observed associations towards the null and potentially underestimate the true effect of air pollution on AKI risk. On the other hand, the use of nationwide health registry data ensures near-complete coverage of hospitalizations and minimizes selection bias. However, the use of the GEM-AQ model should also be considered a strength of the study, as it provides high spatial and temporal resolution and allows for more accurate exposure estimation across the entire study area. Importantly, this approach allows for overcoming the limitations associated with the relatively sparse network of air quality monitoring stations.

## 5. Conclusions

The mid-term exposure to air pollution is associated with an increased risk of AKI-related hospitalisations, with NO_2_ emerging as the most detrimental pollutant. The lack of effect modification across sex and age groups suggests a broadly shared susceptibility at the population level. Importantly, this study extends the current evidence base by identifying mid-term exposure as a relevant temporal window, complementing existing data on short- and long-term effects. Within the broader exposome framework, our findings highlight air pollution as a relevant and potentially modifiable environmental determinant of AKI, underscoring the need to incorporate environmental risk factors into both clinical risk assessment and public health strategies.

## Figures and Tables

**Table 1:** Characteristics of the study population and area.

All Participants (*n* = 47,467)	
Age, Median (IQR)	77 (68–84)
Female, %	51.2 (*n* = 24,303)
Population (people)	8,057,347
Density (per 1 km^2^)	88.5
GDP (USD per capita)	32,107.8
BaP ng/m^3^ (SD)	1.8 (0.8)
PM_2.5_ μg/m^3^ (SD)	21.4 (5.2)
PM_10_ μg/m^3^ (SD)	28.7 (5.7)
NO_2_ μg/m^3^ (SD)	7.5 (1.8)
SO_2_ μg/m^3^ (SD)	3.6 (1.0)
CO μg/m^3^ (SD)	273.6 (15.4)

Abbreviations: BaP, Benzo(a)pyrene; NO_2_, nitrogen dioxide; PM_2.5_, particulate matter with a diameter of 2.5 μm or less; PM_10_, particulate matter with a diameter of 10 μm or less; SO_2_, sulfur dioxide; GDP, gross domestic product (average value for 2011–2020); SD, standard deviation; IQR, interquartile range.

**Table 2:** Yearly data on air pollution conditions.

Year	PM_2.5_, M (SD); μg/m^3^	PM_10_, M (SD); μg/m^3^	NO_2_, M (SD); μg/m^3^	CO, M (SD); μg/m^3^	SO_2_, M (SD); μg/m^3^	BaP, M (SD); ng/m^3^
2011	28.8 (7.5)	21.4 (4.2)	9.5 (2.1)	258.1 (19.6)	5.8 (1.5)	1.7 (0.8)
2012	26.6 (6.9)	20.6 (3.9)	8.9 (2.1)	257.0 (19.0)	6.7 (1.6)	4.2 (1.9)
2013	23.4 (6.3)	22.9 (4.4)	8.1 (2.2)	329.5 (18.9)	4.3 (1.4)	1.6 (0.8)
2014	21.5 (5.2)	29.4 (6.6)	6.2 (1.5)	269.9 (11.7)	3.2 (0.9)	1.5 (0.7)
2015	21.3 (6.0)	28.6 (6.8)	6.3 (1.6)	267.9 (14.5)	2.6 (0.9)	1.8 (0.8)
2016	21.5 (5.9)	28.5 (6.7)	6.8 (1.7)	267.9 (14.5)	2.6 (0.9)	1.8 (0.8)
2017	21.9 (5.8)	29.0 (5.9)	7.7 (2.0)	276.6 (19.9)	3.5 (1.1)	1.8 (1.2)
2018	17.2 (3.2)	30.9 (7.1)	7.9 (1.9)	274.9 (13.8)	2.8 (0.9)	1.5 (0.7)
2019	15.5 (2.8)	35.4 (7.8)	6.6 (1.5)	261.1 (10.3)	2.2 (0.6)	1.2 (0.6)
2020	16.1 (3)	38.1 (8.6)	7.1 (1.6)	275.2 (11.3)	2.1 (0.6)	1.2 (0.6)
*p*-value	<0.001					
**Total**	21.4 (5.2)	28.5 (5.7)	7.5 (1.8)	273.6 (15.4)	3.6 (1.0)	1.8 (0.8)

Abbreviations: BaP, Benzo(a)pyrene; NO_2_, nitrogen dioxide; PM_2.5_, particulate matter with a diameter of 2.5 μm or less; PM_10_, particulate matter with a diameter of 10 μm or less; SO_2_, sulfur dioxide; SD, standard deviation.

## Data Availability

Anonymized data that support the findings of this study are available based on reasonable requests.

## Abbreviations

The following abbreviations are used in this manuscript:

AKI	acute kidney injury
CKD	chronic kidney disease
BaP	Benzo(a)pyrene
NO_2_	nitrogen dioxide
SO_2_	sulfur dioxide
PM_2.5_	particulate matter with a diameter of 2.5 μm or less
PM_10_	particulate matter with a diameter of 10 μm or less
IQR	interquartile range
RR	risk ratio
OR	odds ratio
CI	confidence interval
SD	standard deviation
GDP	gross domestic product
USD	United States dollar
